# A simple and fast technique to cannulate the contralateral gate

**DOI:** 10.1016/j.jvscit.2025.101991

**Published:** 2025-09-24

**Authors:** Pekka Aho, Patrick Björkman, Sani Laukontaus, Maarit Venermo

**Affiliations:** Department of Vascular Surgery, Abdominal Center, University of Helsinki and Helsinki University Hospital, Helsinki, Finland

**Keywords:** Endovascular aneurysm repair, Endovascular technique, Aneurysm

## Abstract

In challenging anatomy and large aneurysms, cannulation of the contralateral gate may be time-consuming and sometimes requires crossover approach with a snare. At Helsinki University Hospital, we have since 2016 used the so-called side-rail technique. This technique is described in detail in this paper including video material on the performance of the procedure. We have found this simple technique to be time and radiation sparing, and we now use it routinely in most endovascular aortic repair procedures.

In endovascular aortic repair (EVAR) of large abdominal aortic aneurysms (AAAs), the anatomy may be challenging, leading to time-consuming cannulation of the contralateral gate. Especially when the aorta is elongated and tortuous, the contralateral gate can point in an unfavorable direction, leading to a long distance between the guiding catheter and the contralateral gate inside the large aneurysm sac.^1^ In addition to using different guiding catheters, one approach is to go crossover and snare the wire to the contralateral side.^2^ This, however, can be difficult or even impossible in ruptured AAAs if an occlusion balloon is deployed inside the main body. Crossover cannulation can be avoided following simple, and often useful, quick steps that we call the “side-rail technique.”

## Technique

In the side-rail technique, after insertion of the contralateral introducer sheath, the stiff guidewire is left in place, and an angiography catheter is inserted parallel to the stiff wire. The main body is deployed so that the contralateral gate opens on top of the contralateral stiff wire. Angiography and, later, the cannulation of the contralateral limb are performed using the pigtail (or another catheter) without removing the stiff guidewire from the aorta (Fig). The contralateral stiff guidewire is now outside the main body, but parallel to the contralateral gate. The contralateral sheath is brought close to the contralateral gate along the stiff wire (Fig). This way, cannulation through the pigtail is easily facilitated without the need for extensive catheter exchanges (Fig). We usually use large 12F to 16F DrySeal sheaths (W.L. Gore) for EVAR procedures. However, the only requirement for the contralateral sheath is to be long enough to reach the aorta and large enough to fit a 0.035″ guidewire parallel to a 4F or 5F pigtail. After cannulation, the stiff wire between the graft and aneurysm neck can be removed and inserted through the cannulated graft, and the contralateral limb inserted. The Supplementary Video (online only) demonstrates the procedure in an elective EVAR.

**Fig:**
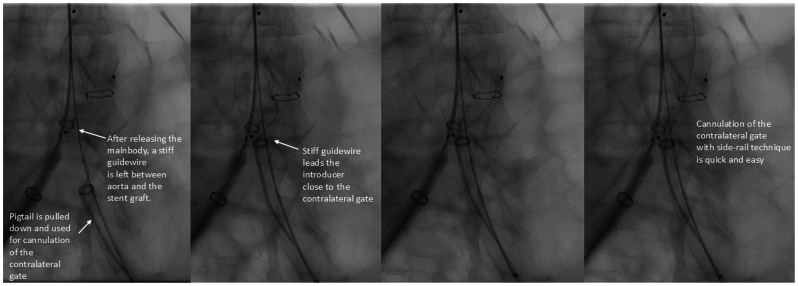
Side-rail technique for cannulation of the contralateral gate.

## Discussion

Contralateral gate cannulation, when difficult, may significantly prolong the EVAR procedure. In elective cases, this may not cause any major damage to a patient, although operation room time is valuable. However, in ruptured cases, faster aneurysm exclusion limits blood loss. Even if an occlusion balloon is deployed below the renal arteries and the patient is not in immediate risk of death due to prolonging cannulation time, backbleeding from the iliac arteries may increase the size of the hematoma and consequently the risk of abdominal compartment syndrome. Thus, every effort should be made to minimize the time to aneurysm exclusion. Expediting the cannulation time is therefore significant. In the current paper and videos, we demonstrate a so-called side-rail technique, which, in our experience, shortens the cannulation time significantly. Although we have not conducted a formal comparison of cannulation times between the side-rail technique and conventional methods, the side-rail technique has, due to its simplicity and ease, been readily embraced by all surgeons in our clinic performing EVAR.

Many grafts, such as the Medtronic Endurant II and Cook Zenith grafts, can by their instructions for use be deployed without additional large sheaths. However, with the former, it is our standard practice to use additional sheaths as you will have to balloon the overlap zones anyway in the end. Furthermore, it is part of our rupture protocol to initially introduce large sheaths bilaterally in order to have flexibility and possibilities when performing the EVAR with an occlusion balloon in place.

There are several other techniques facilitating gate cannulation. Traditionally, shaped catheters have been used for this purpose, and steerable sheaths can also be very useful. Furthermore, going up and over from the ipsilateral side or coming down from above through arm access and snaring the wire from the contralateral side are practical options. However, one of the main advantages of the side-rail technique is that usually no extra equipment or tools are required. Earlier, some papers were published describing techniques that may reduce cannulation time in complex cases. Lee et al^3^ presented a technique where the contralateral guidewire is cannulated into the ipsilateral long limb, the ipsilateral guidewire is cannulated into the contralateral short limb, and the limbs are deployed in a “ballerina” position. This technique requires deployment of both limbs of the main body into the aneurysm sac and thus is not ideal in patients with rupture, where sealing should be achieved as soon as possible, preferably with two components, main body and limb, and thus, the ipsilateral limb of the main body is extended to the common iliac artery. Pakeliani et al^4^ published a paper in which they describe a technique that is similar to our side-rail technique. In addition to the technical description, we provide several videos on the use of this technique.

## Conclusions

We have found this simple time- and radiation-saving technique to be very useful, especially in large, tortuous AAA ruptures. We use it routinely in most EVARs, both elective and emergency.

## Funding

None.

## Disclosures

M.V. has been paid consulting fees by Isomap. P.B. has been paid consulting and proctoring fees by Cook and W.L. Gore. The remaining authors report no conflicts.

## Appendix (online only)

Supplementary Video Cannulation of the contralateral gate using the side-rail technique.

## References

[1] Dang W., Kilian M., Peterson M.D., Cinà C. (2010). Relationship between access side used to deliver the main body of bifurcated prostheses for endovascular aneurysm repair and speed of cannulation of the contralateral limb. J Vasc Surg.

[2] Zeng Q., Huang L., Huang X., Peng M. (2015). Endovascular repair of abdominal aortic aneurysm with severely angulated neck and tortuous artery access: case report and literature review. BMC Surg.

[3] Lee P.Y., Chen P.L., Shih C.C., Chen I.M. (2019). Cross-wire technique for difficult contralateral limb cannulation during endovascular abdominal aneurysm repair for tortuous proximal aortic neck. Interact Cardiovasc Thorac Surg.

[4] Pakeliani D., Lachat M., Blohmé L. (2020). Improved technique for sheath supported contralateral limb gate cannulation in endovascular abdominal aortic aneurysm repair. Vasa.

